# Minimally Invasive Anatomic Liver Resection for Hepatocellular Carcinoma Using the Extrahepatic Glissonian Approach: Surgical Techniques and Comparison of Outcomes with the Open Approach and between the Laparoscopic and Robotic Approaches

**DOI:** 10.3390/cancers15082219

**Published:** 2023-04-09

**Authors:** Yutaro Kato, Atsushi Sugioka, Masayuki Kojima, Satoshi Mii, Yuichiro Uchida, Hideaki Iwama, Takuya Mizumoto, Takeshi Takahara, Ichiro Uyama

**Affiliations:** 1 Department of Advanced Robotic and Endoscopic Surgery, Fujita Health University, Toyoake 470-1192, Japan; iuyama@fujita-hu.ac.jp; 2 International Medical Center, Fujita Health University Hospital, Toyoake 470-1192, Japan; sugioka@fujita-hu.ac.jp; 3 Department of Surgery, Fujita Health University, Toyoake 470-1192, Japan; kojima@fujita-hu.ac.jp (M.K.); smii@fujita-hu.ac.jp (S.M.); yuichiro.uchida@fujita-hu.ac.jp (Y.U.); hideaki.iwama@fujita-hu.ac.jp (H.I.); takuya.mizumoto@fujita-hu.ac.jp (T.M.); takeshi.takahara@fujita-hu.ac.jp (T.T.)

**Keywords:** minimally invasive liver resection, anatomic liver resection, robotic liver resection, laparoscopic liver resection, Glissonian approach, hepatocellular carcinoma

## Abstract

**Simple Summary:**

Surgical techniques and outcomes of minimally invasive anatomic liver resection (AR) for hepatocellular carcinoma (HCC) are undefined. In 327 HCC patients undergoing 185 open (OAR) and 142 minimally invasive (MIAR; 102 laparoscopic and 40 robotic) ARs, perioperative and long-term outcomes were compared, using propensity score matching. After matching (91:91), compared to OAR, MIAR was significantly associated with longer operative time; less blood loss; a lower transfusion rate; lower rates of 90-day major morbidity, bile leak or collection, and 90-day mortality; and shorter hospital stay. On the other hand, laparoscopic and robotic AR cohorts after matching (31:31) had comparable perioperative outcomes. Postoperative overall and recurrence-free survivals of newly developed HCC were comparable between OAR and MIAR or between laparoscopic and robotic cases. MIAR was technically standardized using the extrahepatic Glissonian approach. MIAR was safe, feasible, and oncologically acceptable and would be the first choice of AR in selected HCC patients.

**Abstract:**

Surgical techniques and outcomes of minimally invasive anatomic liver resection (AR) using the extrahepatic Glissonian approach for hepatocellular carcinoma (HCC) are undefined. In 327 HCC cases undergoing 185 open (OAR) and 142 minimally invasive (MIAR; 102 laparoscopic and 40 robotic) ARs, perioperative and long-term outcomes were compared between the approaches, using propensity score matching. After matching (91:91), compared to OAR, MIAR was significantly associated with longer operative time (643 vs. 579 min, *p* = 0.028); less blood loss (274 vs. 955 g, *p* < 0.0001); a lower transfusion rate (17.6% vs. 47.3%, *p* < 0.0001); lower rates of major 90-day morbidity (4.4% vs. 20.9%, *p* = 0.0008), bile leak or collection (1.1% vs. 11.0%, *p* = 0.005), and 90-day mortality (0% vs. 4.4%, *p* = 0.043); and shorter hospital stay (15 vs. 29 days, *p* < 0.0001). On the other hand, laparoscopic and robotic AR cohorts after matching (31:31) had comparable perioperative outcomes. Overall and recurrence-free survivals after AR for newly developed HCC were comparable between OAR and MIAR, with potentially improved survivals in MIAR. The survivals were comparable between laparoscopic and robotic AR. MIAR was technically standardized using the extrahepatic Glissonian approach. MIAR was safe, feasible, and oncologically acceptable and would be the first choice of AR in selected HCC patients.

## 1. Introduction

Anatomic liver resection (AR) is a hepatectomy procedure, where an anatomically determined liver territory that is supplied by the arbitrary Glissonian or portal pedicles (GPs) is completely and optimally resected. Therefore, AR includes both major liver resection of three or more Couinaud’s segments as well as parenchyma-preserving resection represented by mono- or bi- (sub)segmentectomy and monosectionectomy, and their combinatory resection. AR is expected to achieve both high curability and functional safety in liver resection for malignancy. In particular, parenchyma-sparing AR (PSAR), such as isolated segmentectomy and sectionectomy, is considered to confer benefits for resection of hepatocellular carcinoma (HCC), which is characterized by intra-portal vein tumor spread and accompanying impaired hepatic functional reserve [1,2,3].

The safety and feasibility of minimally invasive AR (MIAR) has been partly established in previous studies [4,5,6,7,8,9], though MIAR, particularly PSAR, is still not technically standardized and is regarded as a group of procedures suitable for expert hands or for high volume centers [5,6,8]. Furthermore, perioperative and long-term outcomes of MIAR have not been fully studied nor have they been compared to those of open AR (OAR) in detail. Surgical HCC patients have been shown to obtain oncological benefits from AR, if technically and functionally applicable, compared to non-anatomic liver resection (NAR) [2,3,9]. Therefore, technical standardization of MIAR and evaluation of its surgical results in HCC patients in comparison to those of OAR, can serve to reinforce or revise surgical strategies for HCC.

In this single-center study on 327 consecutive AR cases of HCC, including 185 OAR and 142 MIAR cases, we present our standardized surgical techniques for MIAR and compare perioperative outcomes between OAR and MIAR, as well as between laparoscopic and robotic AR, using propensity score matching (PSM) analyses. In addition, long-term outcomes after AR for newly developed HCC were compared between OAR and MIAR, as well as between laparoscopic and robotic AR. Based on the results, we discuss the technical, surgical, and oncologic aspects of MIAR in surgical management of HCC.

## 2. Materials and Methods

### 2.1. Terminology and Definition of AR

The terminology for liver anatomy and procedures of hepatectomy was primarily based on the Brisbane 2000 Terminology of Liver Anatomy and Resections [10] and Couinaud’s classification [11]. Additionally, the anatomic subsegmentectomy was determined according to the PAM-meeting classification [12]. Thus, AR included trisectionectomy, bisectionectomy including right or left hemihepatectomy and central bisectionectomy, monosectionectomy, (sub)segmentectomy, and their continuous combination. Anatomic subsegmentectomy was defined as the resection of an isolated liver territory that is supplied by the third (or fourth) order division GPs or by its combination with the adjacent GPs smaller than sectional GPs. Isolated total caudate lobectomy was included in the segmentectomy and resection of the Spiegel lobe by dividing the left caudate GP at its root and was defined as subsegmentectomy [13]. Left lateral sectionectomy was included in sectionectomy because it was performed using the extrahepatic Glissonian approach, where GPs to Sg2 and Sg3 were isolated and divided extrahepatically before starting parenchymal dissection. In this study, we excluded AR cases with biliary or vascular reconstruction, those with concomitant extrahepatic procedures, and live donor hepatectomy.

### 2.2. Surgical Indications for MIAR for HCC

At our institution, AR was the first choice of hepatectomy procedure for HCC, if appropriate and applicable, irrespective of the surgical approaches; open, laparoscopic, or robotic. Selection of AR and the type of hepatectomy were based on the location, number, and size of tumors as well as patients’ hepatic functional reserve, mostly according to the so-called Makuuchi criteria, and physical status [14].

Selection of OAR or MIAR was based on the operative difficulties depending on the tumor and patient characteristics (Ban’s criteria) [15] and the surgeons’ capability. It was also dependent on the chronological era, social background, and surgeons’ preference. Basic indications for MIAR were in accordance with the following conditions: (1) tumors with a diameter ≤15 cm, without limitation of tumor location; (2) five or fewer excision sites; and (3) no need for biliary or vascular reconstruction. Several carefully selected cases with reconstructive procedures were performed on a clinical-study-based practice.

Selection of the surgical approaches was greatly affected by the medical paying system. Until 2015, before the start of national insurance coverage of MIAR in our country, the open approach was the first choice for AR in most patients, while from 2016, the laparoscopic approach had the priority, if indicated. Until March 2022, robotic liver resection was a practice at patients’ own expense in Japan, which significantly affected selection of the laparoscopic or robotic approach. After starting reimbursement of robotic liver resection from April 2022, robotic AR was selected at the surgeons’ or patients’ preferences as well as depending on the machine availability in the hospital.

### 2.3. Baseline Data Collection

Patient, tumor, and surgical baseline data were retrospectively collected from the patients’ medical charts. The patient baseline data included age, sex, body mass index (BMI), American Society of Anesthesiology—Performance Status (ASA) score, presence of diabetes, serum biomarkers, etiology of background liver disease (hepatitis B (HBV), hepatitis C (HCV) or non-B and non-C (NBNC)), indocyanine green (ICG) retention rate at 15 min (ICGR15), Child–Pugh grade, and histologically proven cirrhosis (postoperative evaluation). Tumor characteristics included location, number and size of the tumors, tumor stages according to the classification of the Liver Cancer Study Group of Japan [16], and serum tumor markers (alpha-fetoprotein (AFP); des-gamma-carboxy prothrombin (DCP)). Posterosuperior (PS) segments were defined as segments Sg1, Sg4a, Sg7, and Sg8 and were regarded as “locally difficult segments”. The other segments were classified as anterolateral (AL) segments. Pathologic tumor stages and differentiation grades were determined postoperatively. The surgical baseline data included types of AR (left lateral sectionectomy, segmentectomy, sectionectomy, hemihepatectomy, or trisectionectomy) and repeat hepatectomy.

### 2.4. Surgical Techniques for AR

The surgical techniques of AR described below were consistently used either in OAR or MIAR and in any types of AR, irrespective of location of the target anatomic liver territory [5,6,17,18,19]. Inflow control was based on the extrahepatic Glissonian approach, where GPs to the target territory to be resected were first isolated and clamped or divided extrahepatically before parenchymal dissection was started.

Then, the optimal amount of parenchyma was resected along the demarcation line or the intersegmental plane that was visualized by macroscopic discoloration or by the ICG negative staining method [5,6,17]. During parenchymal dissection, the landmark hepatic veins (HVs) were exposed from their root side in the cranial-to-caudal direction (HV root-at first one-way parenchymal dissection). The extrahepatic Glissonian approach and HV root-at first one-way parenchymal dissection were facilitated by the anatomical background of Laennec’s capsule at the hilum and major hepatic veins, as well as the hilar ‘Gate theory’, as described previously [6,13,18,19].

**Figure 1:**
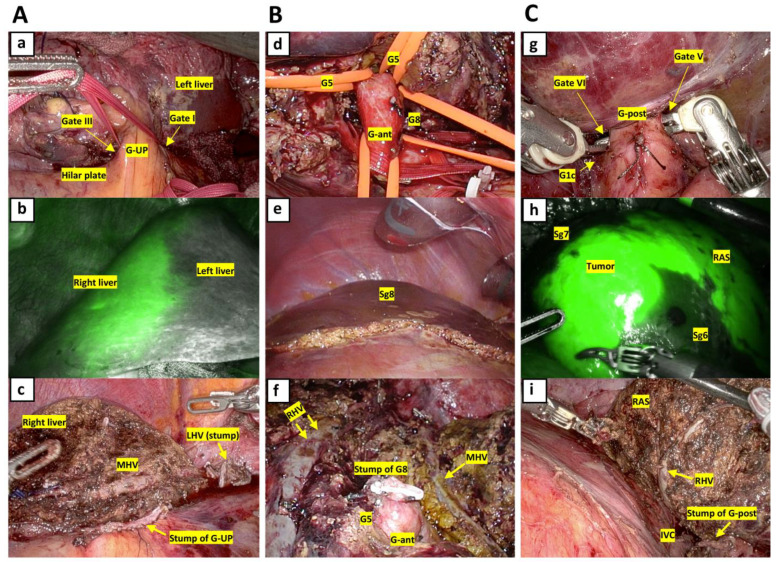
Anatomic liver resection using the extrahepatic Glissonian approach. (**A**) robotic left hemihepatectomy: (**a**) extrahepatic isolation of left Glissonian pedicle above the hilar plate (G-UP) by passing a tape between Gates I and III; (**b**) demarcation line between the right and left liver, using Firefly mode; (**c**) completion of left hemihepatectomy showing the exposed middle hepatic vein (MHV) and resected stumps of G-UP and left hepatic vein (LHV); (**B**) laparoscopic segmentectomy 8; (**d**) extrahepatic isolation of Glissonian pedicles of right anterior section (G-ant), Sg5 (G5), and Sg8 (G8); (**e**): selective isolated ischemia of Sg8; (**f**) completion of segmentectomy 8 showing the exposed MHV and right hepatic vein (RHV) and resected stump of G8; (**C**) robotic right posterior sectionectomy; (**g**) extrahepatic isolation of Glissonian pedicle of right posterior section (G-post); note Gates V and VI and caudate process pedicle (G1c); (**h**): Firefly mode after clamping G-post and intravenous ICG injection; note positive staining of the tumor and right anterior section (RAS) and negative staining of Sg6 and Sg7; (**i**) completion of right posterior sectionectomy showing the exposed RHV, inferior vena cava (IVC), and the resected stump of G-post.

Several examples of the extrahepatic Glissonian approach to isolate the target GPs for MIAR are described in Figure 1. In left hemihepatectomy (Figure 1A), extrahepatic isolation of the left Glissonian pedicle above the hilar plate (G-UP) was facilitated by dissecting Gates I and III and passing forceps and a tape between the gates (Figure 1Aa). In isolated segmentectomy 8 (Figure 1B), to isolate the target GP of Sg8 (G8) extrahepatically (Figure 1Ba), the right anterior section GP (G-ant) was first isolated after cystic plate cholecystectomy in which the cystic plate was resected along with the gall bladder. By exerting traction of G-ant downward, we isolated Sg5 GP (G5) extrahepatically, and finally, G8 was isolated extrahepatically, using the tape switching method (subtraction method) [6,13,19]. In right posterior sectionectomy (Figure 1C), the GP of the posterior section (G-post) was isolated extrahepatically by passing a tape between Gates V and VI (Figure 1Cg). Through selective occlusion of these target pedicles, the isolated ischemic anatomic territories were identified macroscopically as discolored areas or by using an ICG negative staining technique (Figure 1A(b),B(e),C(h)).

Selective occlusion of inflow to the anatomic territory to be resected was followed by HV root-at first one-way parenchymal dissection, during which the already clamped target pedicles were exposed and divided on the liver dissecting plane. For parenchymal dissection, Cavitron Ultrasonic Surgical Aspirator (CUSA^®^) was used in open and laparoscopic cases, and a clamp-crushing method and ultrasonic coagulating shears were used in robotic cases. Pringle maneuver was not used routinely but was applied on demand. Parenchyma was dissected in the cranial-to-caudal direction, and AR was completed (Figure 1A(c),B(f),C(i)).

### 2.5. Perioperative Data

Intraoperative outcomes were evaluated by operative time, blood loss, liver parenchymal dissection time, transfusion (of any blood elements), use of the Pringle maneuver, and open conversion (in MIAR). Postoperative outcomes were evaluated by the serum maximum levels of maximum total bilirubin (TB), aspartate aminotransferase (AST) and C-reactive protein (CRP), the serum minimum level of prothrombin time (PT), morbidity graded according to the Clavien–Dindo (C–D) classification [20], mortality, R0 resection, and the length of postoperative hospital stay (LOS). Overall and major complications were defined as those within 90 postoperative days of any C–D grades and those of ≥C–D grade IIIa, respectively.

### 2.6. Statistical Analysis

Continuous data were expressed as median with range (baseline data) or interquartile range (perioperative data) and compared using the Kruskal–Wallis test. Categorical data were compared using the Pearson’s chi-square test. In some comparative studies, 1:1 PSM was conducted to reduce biases. In comparison of perioperative outcomes between the OAR and MIAR cohorts, the following 11 variables were matched for PSM: age, sex, ASA class (I or II/≥III), ICGR15 (<13.0%/≥13.0%), etiology of underlying liver disease (HBV/HCV/NBNC), tumor number (single/multiple), tumor size (<4.0/≥4.0, cm), tumor location (AL/PS), tumor stage (I or II/≥III), types of resection (left lateral sectionectomy/segmentectomy/sectionectomy/≥hemihepatectomy), and previous hepatectomy (yes/no). In comparison of perioperative outcomes between the laparoscopic and robotic cohorts, age, sex, presence of cirrhosis (yes/no), tumor number (single/multiple), tumor size (<3.0/≥3.0, cm), types of resection, and previous hepatectomy (yes/no) were matched. In analyses of long-term outcomes, we examined newly developed HCC cases only.

In comparison of long-term outcomes between the OAR and MIAR cohorts, 11 variables, including age, sex, ASA class, etiology, presence of cirrhosis, ICGR15 (<13.0%/≥13.0%), tumor number (single/multiple), tumor size (<4.0/≥4.0, cm), tumor stage, pathological tumor differentiation, and types of resection, were matched to reduce biases. Long-term outcomes were also compared between the unmatched and matched laparoscopic and robotic AR cohorts. In this analysis, age, sex, tumor size (<4.0/≥4.0, cm), tumor number (single/multiple), and presence of histology-proven cirrhosis (yes/no) were matched.

The PSM method was the nearest neighborhood method with a caliper width of 0.20. The standard mean deviation (SMD) was calculated for all studied variables, and an SMD <0.20 was confirmed for all matched variables, which indicated appropriate matching. The postoperative overall survival (OS) and recurrence-free survival (RFS) were analyzed only in patients with newly developed HCC, using the Kaplan–Meier method. *p* <0.050 was considered statistically significant. Statistical analyses were performed using JMP^®^ software ver. 14.0 (SAS Institute, Cary, NC, USA).

## 3. Results

Between 2010 and October 2022, we performed 667 liver resections for HCC, including 306 open, 279 laparoscopic, and 82 robotic resections, at our institution. Among these 667 cases, we retrospectively reviewed the consecutive 327 AR cases, consisting of 185 OAR and 142 MIAR (102 laparoscopic and 40 robotic) cases, where AR using the extrahepatic Glissonian approach was performed without biliary or vascular reconstruction or concomitant resection of extrahepatic organs.

### 3.1. Perioperative Outcomes

#### 3.1.1. Comparison between OAR and MIAR

Patient and Tumor Baseline Data

Patient and tumor baseline data were compared between OAR and MIAR (Table 1). Before PSM (185 OAR and 142 MIAR cases), compared to OAR, MIAR was significantly associated with higher BMI, lower ASA class, lower ICGR15, lower Child–Pugh class, smaller tumor size, lower AFP and DCP levels, and more favorable tumor stage. Further, compared to OAR, MIAR was significantly associated with a lower rate of sectionectomy or more extensive procedures, a lower proportion of major resection, and a higher rate of the repeat hepatectomy setting. After 1:1 PSM (91:91), the OAR and MIAR groups were comparable in terms of all studied variables.

##### Perioperative Outcomes

Comparative perioperative outcomes are shown in Table 2. Before PSM, compared to OAR (n = 185), MIAR (n = 142) was significantly associated with less blood loss, a lower transfusion rate, lower postoperative serum maximum TB and CRP levels, higher maximum AST level, lower overall and major morbidity rates, a lower 90-day mortality rate, and shorter LOS.

After 1:1 PSM (91:91), compared to OAR, MIAR was significantly associated with longer operative time (643 vs. 579 min, *p* = 0.028), less blood loss (275 vs. 955 g, *p* < 0.0001), a lower transfusion rate (17.6% vs. 47.3%, *p* < 0.0001), a lower maximum TB (1.5 vs. 2.2 mg/dL, *p* < 0.0001), higher maximum AST (593 vs. 438 IU/L, *p* = 0.043), and lower maximum CRP (8.62 vs. 11.0 mg/dL, *p* = 0.017) levels. Furthermore, MIAR was significantly associated with lower rates of overall (36.3% vs. 57.1%, *p* = 0.005) and major (4.4% vs. 20.9%, *p* = 0.0008) morbidity, a lower rate of bile leak or collection (1.1% vs. 11.0%, *p* = 0.005), and shorter LOS (15 vs. 29 days, *p* < 0.0001).

#### 3.1.2. Comparison between Laparoscopic and Robotic AR for HCC

In the next set of analyses, we compared baseline data and perioperative outcomes between the two MIAR approaches: laparoscopic and robotic.

##### Patient and Tumor Baseline Data

Baseline data were compared between the laparoscopic and robotic AR groups (Table 3). Before PSM (102 laparoscopic vs. 40 robotic cases), compared to laparoscopic AR, robotic AR was significantly associated with a lower rate of cirrhosis, smaller tumor number and size, a lower AFP level, and a higher rate of repeat hepatectomy setting. After 1:1 PSM (31:31), all studied variables were comparable between the laparoscopic and robotic AR groups.

##### Perioperative Outcomes

Perioperative outcomes are shown in Table 4. Before PSM, laparoscopic and robotic AR groups had comparable outcomes, except for the significantly higher rate of Pringle maneuver application (35.0% vs. 17.7%, *p* = 0.026), the higher maximum AST level (767 vs. 546 IU/L, *p* = 0.026), and the lower minimum PT level (60% vs. 64%, *p* = 0.032) in the robotic AR group. After PSM, both groups had comparable perioperative outcomes.

### 3.2. Long-Term Outcomes after AR for Newly Developed HCC

In the next set of analyses, we studied long-term outcomes after AR in 276 patients with newly developed HCC, who underwent 163 OARs and 113 MIARs (89 laparoscopic and 24 robotic ARs), respectively, and compared OS and RFS between OAR and MIAR and between laparoscopic and robotic AR.

#### 3.2.1. Comparison of Long-Term Outcomes between OAR and MIAR

##### Patient and Tumor Baseline Data

Baseline data were compared between 163 patients undergoing OAR and 113 patients undergoing MIAR for newly developed HCC (Table 5). Before PSM (163:113), compared to OAR, MIAR was significantly associated with higher BMI, a lower ASA class, lower ICGR 15, smaller tumor number and size, more favorable tumor stages, and higher AFP and DCP levels. In addition, types of resection were significantly more extensive, with a higher proportion of major resection in OAR than in MIAR. After 1:1 PSM (76:76), all studied variables were comparable between OAR and MIAR.

##### Long-Term Outcomes

**Figure 2:**
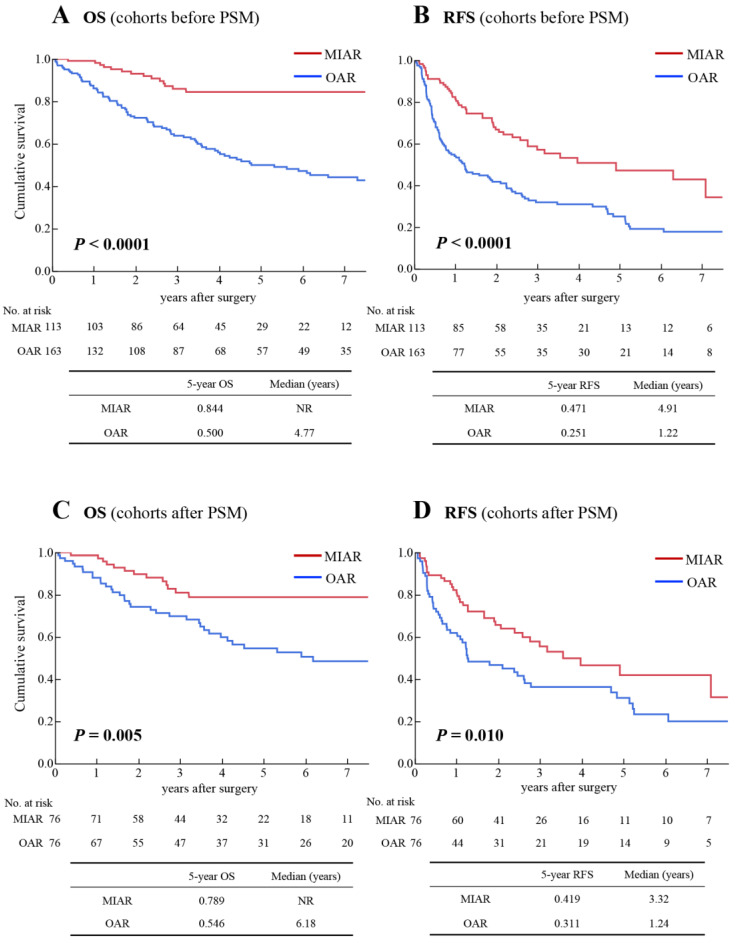
Overall (OS) and recurrence-free (RFS) survivals after OAR and MIAR: (**A**) OS in the comparative cohorts before PSM; (**B**). RFS in the comparative cohorts before PSM; (**C**) OS in the comparative cohorts after PSM; (**D**) RFS in the comparative cohorts after PSM; NR: not reached.

Comparing survival rates between the unmatched patients undergoing MIAR (n = 113) and OAR (n = 163), MIAR patients had statistically longer OS (Figure 2A, *p* < 0.0001; 5-year rate: 84.4% vs. 50.0%) and longer RFS (Figure 2B, *p* < 0.0001; 5-year rate: 47.1% vs. 25.1%) than OAR patients. For the matched cohorts after PSM, compared to OAR (n = 76), MIAR patients (n = 76) were associated with statistically longer OS (Figure 2C, *p* = 0.005; 5-year rate: 78.9% vs. 54.6%) and longer RFS (Figure 2D, *p* = 0.010; 5-year rate: 41.9% vs. 31.1%).

#### 3.2.2. Comparison of Long-Term Outcomes between Laparoscopic and Robotic AR

##### Patient and Tumor Baseline Data

Patient and tumor baseline data were compared between the unmatched 89 laparoscopic AR and 24 robotic AR cases (Table 6). Before PSM, compared to laparoscopic AR, robotic AR was significantly associated with a lower rate of histology-proven cirrhosis (12.5% vs. 42.7%, *p* = 0.006) and a lower rate of tumors ≥4.0 cm (25.0% vs. 48.3%, *p* = 0.041). One-to-one PSM identified 22 laparoscopic and 22 robotic matched cases. After PSM, all evaluated patient and tumor characteristics were comparable between the laparoscopic and robotic AR cohorts.

##### Survival Data

**Figure 3:**
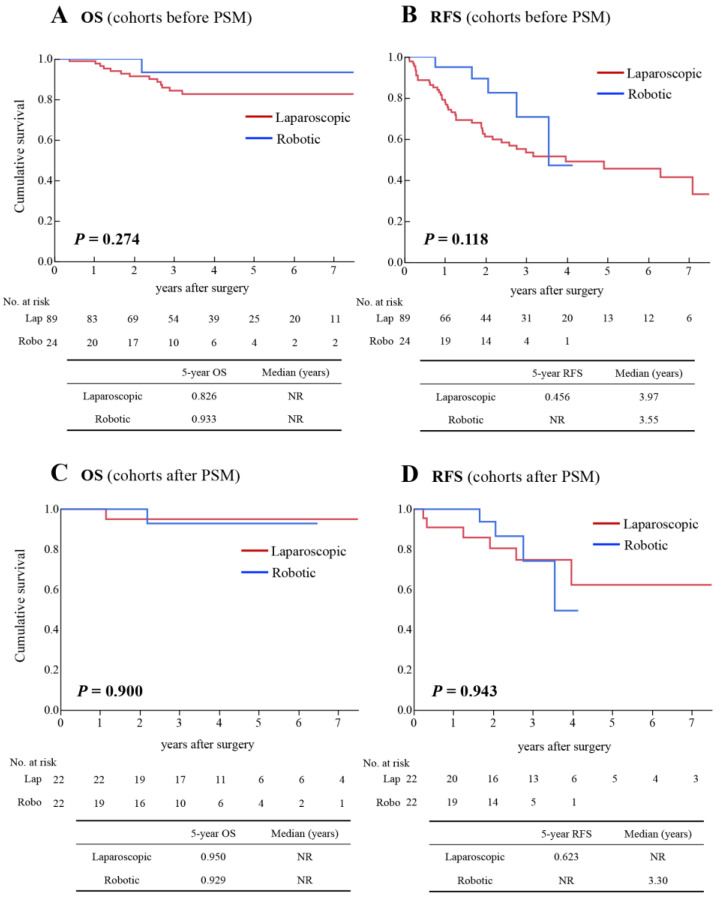
Overall (OS) and recurrence-free (RFS) survivals after laparoscopic and robotic anatomic resection: (**A**) OS in the unmatched cohorts; (**B**) RFS in the unmatched cohorts; (**C**) OS in the matched cohorts; (**D**) RFS in the matched cohorts; NR: not reached.

Comparison between the unmatched cohorts of laparoscopic (n = 89) and robotic (n = 24) approaches showed comparable OS (Figure 3A) and RFS (Figure 3B). Similarly, OS (Figure 3C) and RFS (Figure 3D) were comparable between the matched laparoscopic AR (n = 22) and robotic AR (n = 22) cohorts.

##### Details of Postoperative Recurrence

In the study period, tumor recurrence was observed in 162 (58.7%) of the entire 276 patients, including 111 OAR and 51 MIAR patients; the recurrence rate was significantly lower in the unmatched MIAR than in the OAR cohorts (45.1% vs. 68.1%, *p* < 0.0001). Between the matched OAR and MIAR cohorts (76:76), the recurrence rate was still significantly higher in OAR (n = 50, 65.8%) than in MIAR (n = 37, 48.7%) (*p* = 0.033).

The details of recurrence and its treatments are shown in Table 7. In recurrent cases in the entire cohort, patterns of recurrence (intrahepatic and/or extrahepatic), the rate of extrahepatic recurrence, and recurrent organs were comparable between OAR and MIAR. Compared to OAR, MIAR was associated with significantly longer RFS (median: 15.4 vs. 7.6 months, *p* = 0.007) and a significantly lower rate of the first recurrence within 1 postoperative year (37.3% vs. 62.2%, *p* = 0.003). Pathologic tumor stages were more advanced in recurrent cases in the unmatched OAR cohort (*p* = 0.008), while they were comparable between the matched OAR and MIAR cohorts. Pathologic tumor differentiations were comparable between the recurrent cases in the unmatched and matched cohorts. Perioperative morbidity, which has been suggested to affect tumor recurrence in previous studies [21,22], was comparable between OAR and MIAR, both in the unmatched and matched cohorts.

Regarding the treatments for cancer recurrence, in the unmatched cohorts, compared to OAR, MIAR tended to be associated with the higher rates of recurrent organ resection (45.1% vs. 31.5%, *p* = 0.094) and repeat hepatectomy (46.9% vs. 30.8%, *p* = 0.052). Systemic pharmacological treatment using molecular targeted agents (MTAs), such as sorafenib and lenvatinib, or immunotherapy using immune checkpoint inhibitors (ICIs), such as atezolizumab, were performed at a similar rate between the OAR and MIAR cohorts with recurrence (17.7% vs. 17.1%). On the other hand, in the matched cohorts, patterns of recurrence, RFS, timing of the first recurrence, and the rate of resection or systemic pharmacological treatment for recurrent tumors were comparable between OAR and MIAR (Table 7).

##### Times of Surgery and Associated Factors

**Figure 4:**
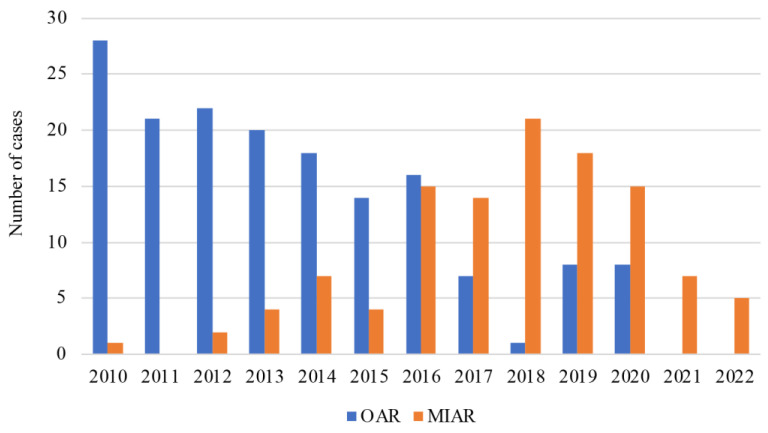
Annual number of cases undergoing OAR (blue bars) and MIAR (orange bars) for newly developed HCC.

To further investigate factors that potentially affect the long-term outcomes after resection of newly developed HCC, we studied the times or era of surgery because significant changes occurred socially in the strategies of HCC treatment as well as in the control of HCV using direct acting antivirals (DAAs) during the study period. We first examined the trends of application of OAR or MIAR throughout the study period (Figure 4). As shown, the ratio of OAR cases in all AR cases significantly decreased, and OAR was gradually replaced by MIAR over the years (*p* < 0.0001). Further, when the study period was divided into the early (2010–2015) and late (2016–2022) periods, MIAR was significantly more frequently performed than OAR in the late period in a comparison between the unmatched (n = 95 (84.1%) vs. n = 40 (24.5%), *p* < 0.0001) and matched (n = 61 (80.3%) vs. n = 14 (18.4%), *p* < 0.0001) cohorts.

At our institution, eradication of HCV using DAAs became active from around 2016, which was coincidental with a remarkable change in the surgical approach from OAR to MIAR. To exclude the impacts of HCV eradication on postoperative recurrence and hepatic functional status and to study the simple impact of different approaches on survival, we examined the long-term outcomes in 184 AR cases without associated HCV infection, i.e., 55 HBV and 129 NBNC cases. One-to-one PSM identified the 42 OAR and 42 MIAR matched cases from the 107 OAR and 77 MIAR unmatched cases. The survival data showed that OS in MIAR, with a 5-year rate of 85.2% and not-reached median survival, was significantly longer than OS in OAR, with a 5-year rate of 43.3% and the median survival time of 48.9 months (*p* = 0.001). Furthermore, RFS in MIAR, with a 5-year rate of 47.4% and the median time of 40.4 months, was significantly longer than RFS in OAR, with a 5-year rate of 21.8% and a median time of 7.6 months (*p* = 0.004).

## 4. Discussion

In this study, we described our surgical techniques of MIAR, including laparoscopic and robotic AR, and compared perioperative and long-term outcomes in HCC cases between OAR and MIAR as well as between laparoscopic and robotic AR, using PSM-based analyses. Our surgical techniques for AR in all studied cases were consistently based on the extrahepatic Glissonian approach and HV root-at first one-way parenchymal dissection, irrespective of the open, laparoscopic, or robotic approach. Furthermore, AR was primarily performed and managed only by surgeons with appropriate skills at our institution. These study settings may have reduced biases, such as surgical techniques and surgeons’ experience, which were latent in previous comparative studies.

In the current study, we first audited perioperative outcomes in 142 MIAR cases and compared them to 185 OAR cases. A PSM (91:91)-based analyses showed that compared to OAR, MIAR was significantly associated with less blood loss, a lower transfusion rate, lower postoperative TB and CRP levels, lower rates of 90-day overall and major morbidity and bile leak or intraabdominal collection, a lower rate of 90-day mortality, and shorter LOS. On the other hand, a significantly higher postoperative AST level was observed in the MIAR compared to the OAR group. Next, we compared perioperative outcomes between matched laparoscopic and robotic AR cohorts (46:46), which showed comparable results. Finally, we studied the long-term outcomes after AR for newly developed HCC. PSM-based analyses matching patient and tumor characteristics and types of resection showed comparable or potentially superior OS and RFS in the MIAR cohort compared to those in the OAR cohort, as well as comparable outcomes between the laparoscopic and robotic approaches.

As for technical aspects of MIAR, we believe, from the current and previous results, that we can standardize MIAR by using the extrahepatic Glissonian approach and HV root-at first one-way parenchymal dissection [4,5,6,13,18,19,23]. Furthermore, the consistent usage of these standardized techniques, irrespective of OAR and MIAR, may reduce technical biases, being an advantage of this study setting. These techniques for AR, which were originally developed in OAR at our institution, have been safely and effectively translated into MIAR with acceptable curability, as shown by the favorable perioperative and long-term outcomes. In addition, as the most recent surgical platform, robotics was technically applicable to AR with surgical outcomes comparable to those of the laparoscopic approach. The most important advantage of the extrahepatic Glissonian approach for AR is accurate determination of the anatomic liver area to be resected before starting parenchymal dissection, which leads to accurate, safe, and optimal AR. On the other hand, one of the disadvantages of this approach is that although it is applicable in any type of AR for any liver segments, there are cases where extrahepatic isolation of peripheral subsegmental GPs is unsafe or impossible, depending on the liver anatomy. In this study, PSAR accounted for over 80% of OAR or MIAR cases. Although minimally invasive accurate PSARs are more demanding than standard right or left hemihepatectomy [6,24,25], they were technically standardized and performed safely in this series. Therefore, MIAR including PSAR can provide HCC patients with benefits of not only minimally invasiveness but also safety and curability.

Previous studies comparing open and minimally invasive liver resections have shown less blood loss, decreased morbidity, and shorter LOS in the latter [23,26,27,28,29]. Laparoscopic and robotic liver resections were shown to have comparable perioperative outcomes [23,30]. Results of the current study are in line with those of these studies, though the cohort setting was different; very few studies exclusively selected AR cases for the cohort. However, recent large studies have demonstrated advantages of the robotic approach on perioperative outcomes over the laparoscopic approach in selected types of AR [31,32,33].

Several studies have shown comparable long-term outcomes after resection of HCC between the open and minimally invasive approaches [26,28]. Only one study showed a survival advantage of the laparoscopic over the open approach, though significant biases were suggested in the study setting [29]. In our results, surprisingly, long-term outcomes after MIAR for newly developed HCC appeared to be more favorable than those after OAR (Figure 2). Both OS and RFS were statistically longer in the MIAR than in the OAR cohort, not only before, but also after PSM. The longer OS and RFS in the unmatched MIAR cohort could be largely explained by the more favorable tumor characteristics in this cohort represented by lower tumor stages, lower serum tumor markers, the need for less extensive hepatectomy, and a lower rate of early recurrence (Table 5 and Table 7). Furthermore, the better patients’ physical conditions in this cohort represented by the lower ASA scores and ICGR 15 (Table 5) might have beneficial impacts on postoperative survivals. Additionally, a higher rate of resection of recurrent tumors in the MIAR cohort (Table 7) could have contributed to the longer OS.

On the other hand, it is worth noting that even the matched MIAR cohort after PSM had significantly longer OS and RFS than the OAR cohort. To seek the scenarios behind such potentially better survivals in MIAR, we addressed several relevant points. First, we examined the details of recurrence and found that the patterns of recurrence, the incidence of extrahepatic metastasis and pathologic tumor differentiation, and stages were comparable between the cohorts (Table 7). Second, we examined the times or era of application of OAR or MIAR because the drastic changes in the surgical approach from OAR to MIAR at our institution (Figure 4) coincided with the changing trends in the pharmacological treatment for HCC recurrence toward MTAs or ICIs, along with significant success of HCV eradication by using DAAs. Our results showed, however, that these changes in non-surgical factors were comparable between the matched OAR and MIAR cohorts. Furthermore, OS and RFS in the HCC cohort excluding HCV-infected patients were still longer in MIAR than in OAR, suggesting that HCV eradication had minor impacts on the survival differences between MIAR and OAR in this study.

Other factors potentially contributing to the more favorable RFS in MIAR include theoretical immunological advantages conferred by its lower invasiveness. The advantages can stem from factors including less physical damage, less disturbance in the perioperative metabolic and nutritional status, and the resultant early recovery after surgery. In our results, such lower invasiveness in MIAR was evidenced by less blood loss, the lower levels of postoperative TB and CRP, and shorter LOS, compared to OAR (Table 2). Furthermore, lower rates of perioperative morbidity and blood transfusion in MIAR (Table 2) may have had beneficial impacts on its long-term outcomes, in line with previous studies on resected cancers including HCC [21,22,34,35].

Another point to be taken into consideration is that in most previous studies comparing open and minimally invasive hepatectomy, both AR and NAR were included. Since NAR tended to be performed for HCC patients with impaired hepatic functional reserve, inclusion of both AR and NAR cases in the study cohorts and the case number ratio of each cohort may have affected the entire comparative survival data on which both oncologic and hepatic functional factors had significant impacts.

Collectively, our data were not enough to explain the differences in the long-term outcomes between the matched OAR and MIAR cohorts, and in the first place, the relatively small sample size in each cohort precludes definite conclusions. Nonetheless, our results suggest that the long-term outcomes after AR for newly developed HCC were at least comparable between OAR and MIAR. Larger studies incorporating more evaluation facets, such as post-recurrence treatment modalities, patient nutritional status and immunity characteristics and tumor genetic information, are necessary to investigate the impact of a minimally invasive approach to AR on long-term outcomes of resected HCC.

In view of our results showing mostly better perioperative outcomes in MIAR than in OAR, as well as comparable or potentially more favorable long-term outcomes in MIAR, it is reasonable to suggest that MIAR would be the first choice for a surgical approach to HCC, at least by an expert. On the other hand, despite the expected functional merits of robotics providing surgical dexterity, the robotic approach did not show advantages in surgical outcomes over the laparoscopic approach to AR in this study. However, the sample size was small, particularly in the robotic group, and techniques of robotic liver surgery are still developing. Nonetheless, the robotic platform may have potential advantages in perioperative outcomes in complex anatomic resection, such as less blood loss, decreased open conversion rate, and decreased morbidity, as suggested in other study settings [31,32,33]. Larger studies are warranted to investigate the potential differences in outcomes between laparoscopic and robotic AR.

There are several limitations to this study. First, this is a retrospective, observational, single-center study, though PSM was conducted to reduce biases. Second, the sample sizes in each PSM-matched cohort are relatively small. Third, long-term outcomes should be carefully interpreted because of the abovementioned small sample size, small number of matched variables in PSM-based analyses, and potential biases from ‘difficult-to-match’ factors, including selection of approach, learning curve of techniques, patient immunological status, era-dependent development of DAAs for HCV eradication, and advances in post-recurrence pharmacological therapy for HCC.

## 5. Conclusions

Although MIAR is technically demanding, particularly for HCC, because of potential underlying liver dysfunction, it can be technically standardized by the extrahepatic Glissonian approach and HV root-at first one-way parenchymal dissection. Furthermore, MIAR for HCC was safe, feasible, and oncologically acceptable, with perioperative outcomes mostly superior to those in OAR and with comparable or potentially more favorable long-term outcomes. A laparoscopic or robotic approach would be the first choice for AR in selected surgical HCC patients, at least by experts. Further larger studies are warranted to investigate potential advantages of MIAR for HCC in terms of long-term outcomes, as well as perioperative and long-term advantages of the robotic or laparoscopic approach over the counterpart.

## Figures and Tables

**Table 1:** Comparison of baseline data between open and minimally invasive AR for HCC.

	Before PSM			After PSM		
	OAR (N = 185)	MIAR (N = 142)	*p*	OAR (N = 91)	MIAR (N = 91)	*p*
Age, years	73 (31–91)	71 (11–86)	0.102	72 (43–91)	72 (29–86)	0.570
Sex, M/F	147/38	113/29	0.979	73/18	73/18	1.000
BMI, kg/m^2^	23.0 (14.7–54.0)	23.6 (15.2–36.3)	**0.013**	23.1 (17.2–54.0)	23.8 (16.0–36.3)	0.456
ASA score, I or II/≥III	145/40	130/12	**0.001**	80/11	81/10	0.817
Diabetes, n (%)	71 (38.4)	53 (37.3)	0.846	38 (41.8)	36 (39.6)	0.763
Total bilirubin, mg/dL	0.7 (0.3–6.4)	0.8 (0.2–1.7)	0.097	0.7 (0.3–1.8)	0.7 (0.2–1.7)	0.782
Prothrombin time, %	98 (18–145)	96 (28–129)	0.386	97 (64–145)	95 (63–129)	0.319
Platelet count, ×10^4^/mm^3^	14.9 (1.2–47.2)	15.5 (4.0–42.2)	0.783	14.7 (1.2–47.2)	15.0 (4.6–29.5)	0.809
ICGR15, %	14.2 (0.6–52.6)	11.1 (0.0–68.3)	**0.0002**	14.1 (0.6–41.0)	12.1 (0.0–68.3)	0.072
≥13.0%, n (%)	100 (55.3)	49 (36.6)	**0.001**	49 (53.9)	42 (46.2)	0.299
Child-Pugh class, A/B	177/8	141/1	**0.047**	86/5	90/1	0.097
Etiology, HBV/HCV/NBNC	37/70/78	35/48/59	0.561	19/30/42	20/31/40	0.956
Cirrhosis (histology), n (%)	58 (31.4)	53 (37.3)	0.258	31 (34.1)	34 (37.4)	0.643
Tumor characteristics						
Location, PS (%)/AL	130 (70.3)/55	88 (62.0)/54	0.115	66 (72.5)/25	61 (67.0)/30	0.420
Number	1 (1–23)	1 (1–6)	0.053	1 (1–6)	1 (1–6)	0.254
Single/Multiple	122/63	107/35	0.066	70/21	63/28	0.242
Size, cm	5.0 (1.0–22.0)	3.2 (0.7–16.0)	**<0.0001**	4.0 (1.0–17.7)	3.8 (0.7–16.0)	0.143
≥4.0 cm, n (%)	114 (61.6)	53 (37.3)	**<0.0001**	47 (51.7)	44 (48.4)	0.657
Stage, I or II/≥III	94/91	108/34	**<0.0001**	63/28	59/32	0.528
AFP, ng/mL	15.1 (1.5–1,213,687.0)	6.4 (1.0–149,880.0)	**0.0005**	11.2 (1.5–636,200.0)	6.9 (1.0–149,880.0)	0.135
DCP, mAU/mL	287 (3–538,983)	67 (10–47,453)	**0.0003**	186 (10–159,600)	74 (10–47,453)	0.056
Types of resection, n (%)			**0.004**			0.976
Left lateral sectionectomy	5 (2.7)	8 (5.6)		4 (4.4)	4 (4.4)	
Segmentectomy	77 (41.6)	83 (58.5)		50 (55.0)	47 (51.7)	
Sectionectomy *	56 (30.3)	30 (21.1)		22 (24.2)	24 (26.4)	
≥Hemihepatectomy	47 (25.4)	21 (14.8)		15 (16.5)	16 (17.6)	
Major Hx (≥3 segs), n (%)	56 (30.3)	23 (16.2)	**0.003**	18 (19.8)	17 (18.7)	0.851
Repeat Hx, n (%)	21 (11.4)	29 (20.4)	**0.024**	13 (14.3)	12 (13.2)	0.830

AR: anatomic liver resection; PSM: propensity score matching; continuous variables: median (range); OAR and MIAR: open and minimally invasive anatomic liver resection, respectively; BMI: body mass index; ASA: American Society of Anesthesiology; ICGR15: the indocyanine green retention rate at 15 min; NBNC: non-B and non-C; PS: posterosuperior; AL: anterolateral; AFP: alpha-fetoprotein; DCP: des-gamma-carboxy prothrombin Sectionectomy *: mono- or central bisectionectomy except for left lateral sectionectomy; Hx: hepatectomy; Bold: statistically significant.

**Table 2:** Comparison of perioperative outcomes between open and minimally invasive AR for HCC.

	Before PSM			After PSM		
	OAR (N = 185)	MIAR (N = 142)	*p*	OAR (N = 91)	MIAR (N = 91)	*p*
Operative time, min	591 (498–781)	637 (539–794)	0.128	579 (474–731)	643 (546–797)	**0.028**
Blood loss, g	1083 (575–2006)	244 (115–493)	**<0.0001**	955 (498–1753)	279 (121–524)	**<0.0001**
Transfusion*, n (%)	98 (53.0)	23 (16.2)	**<0.0001**	42 (47.3)	16 (17.6)	**<0.0001**
Pringle maneuver, n (%)	27 (14.6)	32 (22.5)	0.064	16 (17.6)	21 (23.1)	0.357
Open conversion, n (%)	NA	4 (2.8)	NA	NA	2 (2.2)	NA
Laboratory data						
Max TB, mg/dL	2.3 (1.6–3.4)	1.5 (1.2–2.0)	**<0.0001**	2.2 (1.6–3.1)	1.5 (1.2–1.9)	**<0.0001**
Max AST, IU/L	416 (291–808)	598 (315–1026)	**0.005**	438 (305–823)	593 (348–1016)	**0.043**
Min PT, %	63 (54–68)	63 (58–71)	0.093	64 (54–68)	62 (56–71)	0.810
Max CRP, mg/dL	10.40 (7.65–13.04)	9.00 (6.25–13.01)	**0.028**	11.10 (7.80–12.80)	8.62 (6.32–12.60)	**0.017**
Morbidity (≤90 days), n (%)						
Overall (≥CD-I)	97 (52.4)	50 (35.2)	**0.002**	52 (57.1)	33 (36.3)	**0.005**
Major (≥CD-IIIa)	33 (17.8)	12 (8.5)	**0.015**	19 (20.9)	4 (4.4)	**0.0008**
Bile leak or collection	14 (7.6)	6 (4.2)	0.211	10 (11.0)	1 (1.1)	**0.005**
Mortality, n (%)						
≤30 days	2 (1.1)	0 (0)	0.214	1 (1.1)	0 (0)	0.316
≤90 days	5 (2.7)	0 (0)	**0.048**	4 (4.4)	0 (0)	**0.043**
R0 resection, n (%)	179 (96.8)	141 (99.3)	0.116	88 (96.7)	90 (98.9)	0.312
Length of hospital stay, days	28 (20–40)	15 (12–19)	**<0.0001**	29 (21–43)	15 (13–19)	**<0.0001**

Continuous variables: median (interquartile range); AR: anatomic liver resection; PSM: propensity score matching; OAR and MIAR: open and minimally invasive anatomic liver resection, respectively; Transfusion*: any kinds of blood component or product; NA: not applicable; Max and Min: postoperative serum maximum and minimum levels, respectively, TB: total bilirubin; AST: aspartate aminotransferase; PT: prothrombin time; CRP: C-reactive protein, CD: Clavien–Dindo grade; Bold: statistically significant.

**Table 3:** Comparison of baseline data between laparoscopic and robotic AR for HCC.

	Before PSM			After PSM		
	Laparoscopic AR (N = 102)	Robotic AR (N = 40)	*p*	Laparoscopic AR (N = 31)	Robotic AR (N = 31)	*p*
Age, years	70 (11–86)	72 (21–82)	0.353	70 (36–83)	72 (21–82)	0.989
Sex, M/F	80/22	33/7	0.589	25/6	25/6	1.000
BMI, kg/m^2^	23.6 (15.2–36.3)	23.9 (17.9–30.3)	0.895	23.0 (18.0–33.9)	23.9 (17.9–30.3)	0.186
ASA score, I or II/≥III	94/8	36/4	0.678	29/2	28/3	0.641
Total bilirubin, mg/dL	0.8 (0.2–1.7)	0.7 (0.3–1.3)	0.479	0.7 (0.2–1.6))	0.7 (0.3–1.3)	0.854
Prothrombin time, %	96 (63–129)	97 (28–127)	0.701	96 (67–128)	97 (83–127)	0.849
Platelet count, x10^4^/mm^3^	15.7 (4.0–42.2)	15.3 (7.6–23.5)	0.665	15.5 (4.6–29.5)	15.1 (9.1–23.5)	0.961
ICGR15, %	11.1 (0.6–68.3)	10.9 (0.0–30.8)	0.284	10.5 (0.6–27.6)	11.8 (0.0–30.8)	0.554
≥13.0%, n (%)	35 (36.5)	14 (36.8)	0.967	6 (22.2)	13 (43.3)	0.091
Child-Pugh, A/B	101/1	40/0	0.530	31/0	31/0	1.000
Etiology, HBV/HCV/NBNC	24/36/42	11/12/17	0.805	7/11/13	9/8/14	0.247
Cirrhosis (histology), n (%)	46 (45.1)	7 (17.5)	**0.002**	6 (19.4)	7 (22.6)	0.755
Tumor characteristics						
Location, PS (%)/AL	65 (63.7)/37	23 (57.5)/17	0.492	20 (64.5)/11	16 (51.6)/15	0.303
Number	1 (1–4)	1 (1–6)	**0.047**	1 (1–3)	1 (1–6)	0.927
Single/Multiple	81/21	26/14	0.073	23/8	23/8	1.000
Size, cm	3.5 (0.7–16.0)	2.7 (1.2–12.5)	**0.027**	3.0 (1.5–16.0)	3.0 (1.2–12.5)	0.371
≥4.0 cm, n (%)	44 (43.1)	9 (22.5)	**0.022**	12 (38.7)	8 (25.8)	0.277
Stage, I or II/≥III	81/21	27/1	0.135	23/8	21/10	0.576
AFP, ng/mL	8.2 (1.9–149,880.0)	4.2 (1.0–5811.0)	**0.002**	7.1 (2.0–2708.5)	4.5 (1.0–5811.0)	0.113
DCP, mAU/mL	75 (10–47,453)	44 (11–30,899)	0.071	68 (11–47,032)	4.5 (1.0–5811.0)	0.251
Repeat Hx, n (%)	13 (12.8)	16 (40.0)	**0.0003**	6 (19.4)	7 (22.6)	0.755
Types of resection, n (%)			0.775			0.271
Left lateral sectionectomy	6 (5.9)	2 (5.0)		0 (0)	2 (6.5)	
Segmentectomy	58 (56.9)	25 (62.5)		18 (58.1)	20 (64.5)	
Sectionectomy*	21 (20.6)	9 (22.5)		10 (32.3)	5 (16.1)	
≥Hemihepatectomy	17 (16.7)	4 (10.0)		3 (9.7)	4 (12.9)	
Major Hx (≥3 segs), n (%)	19 (18.6)	4 (10.0)	0.838	31 (100)	4 (12.9)	0.719
Iwate criteria, level, n (%)			0.549			0.327
Intermediate	22 (21.6)	12 (30.0)		5 (16.1)	10 (32.3)	
Advanced	51 (50.0)	17 (42.5)		18 (58.1)	14 (45.2)	
Expert	29 (28.4)	11 (27.5)		8 (25.8)	7 (22.6)	
≥Advanced, n (%)	80 (78.4)	28 (70.0)	0.290	26 (83.9)	21 (67.7)	0.138

PSM: propensity score matching; AR: anatomic liver resection; continuous variables: median (range); BMI: body mass index; ASA: American Society of Anesthesiology; ICGR15: the indocyanine green retention rate at 15 min; NBNC: non-B and non-C; PS: posterosuperior; AL: anterolateral; AFP: alpha-fetoprotein; DCP: des-gamma-carboxy prothrombin; Sectionectomy*: mono- or central bisectionectomy except for left lateral sectionectomy; Hx: hepatectomy; Bold: statistically significant.

**Table 4:** Comparison of perioperative outcomes between laparoscopic and robotic AR for HCC.

	Before PSM			After PSM		
	Laparoscopic AR (N = 102)	Robotic AR (N = 40)	*p*	Laparoscopic AR(N = 31)	Robotic AR (N = 31)	*p*
Operative time, min	631 (525–774)	667 (566–893)	0.157	632 (569–732)	642 (564–891)	0.709
Parenchymal dissection time, min	240 (175–325)	273 (177–340)	0.548	248 (177–333)	227 (139–367)	0.906
Blood loss, g	245 (120–488)	200 (98–635)	0.890	227 (90–468)	170 (98–598)	0.989
Transfusion*, n (%)	16 (15.7)	7 (17.5)	0.792	3 (9.7)	6 (19.4)	0.279
Pringle maneuver, n (%)	18 (17.7)	14 (35.0)	**0.026**	5 (16.1)	10 (32.3)	0.138
Open conversion, n (%)	2 (2.0)	2 (5.0)	0.325	0 (0)	1 (3.2)	0.313
Laboratory data						
Max TB, mg/dL	1.5 (1.2–1.9)	1.5 (1.3–2.0)	0.701	1.4 (1.1–1.6)	1.5 (1.2–2.0)	0.073
Max AST, IU/L	546 (296–913)	767 (370–2,100)	**0.026**	593 (315–903)	707 (348–2,796)	0.275
Min PT, %	64 (59–72)	60 (49–71)	**0.032**	62 (58–73)	60 (51–72)	0.135
Max CRP, mg/dL	8.68 (6.32–12.72)	9.64 (5.97–12.18)	0.396	8.44 (6.32–12.6)	10.18 (5.45–14.11)	0.477
Morbidity (≤90 days), n (%)						
Overall (≥CD-I)	37 (36.3)	13 (32.5)	0.672	10 (32.3)	9 (29.0)	0.783
Major (≥CD-IIIa)	7 (6.9)	5 (12.5)	0.277	2 (6.5)	5 (16.1)	0.229
Bile leak or collection	5 (4.9)	1 (2.5)	0.522	2 (6.5)	1 (3.2)	0.554
Mortality, n (%)						
≤30 days	0 (0)	0 (0)	1.000	0 (0)	0 (0)	1.000
≤90 days	0 (0)	0 (0)	1.000	0 (0)	0 (0)	1.000
R0 resection, n (%)	101 (99.0)	40 (100)	0.530	31 (100)	31 (100)	1.000
Length of hospital stay, days	15 (12–19)	15 (11–18)	0.416	14 (11–18)	15 (11–18)	0.965

PSM: propensity score matching; AR: anatomic liver resection; continuous variables: median (interquartile range); Transfusion*: any kinds of blood component or product; Max and Min: postoperative serum maximum and minimum levels, respectively; TB: total bilirubin; AST: aspartate aminotransferase; PT: prothrombin time; CRP: C-reactive protein; CD: Clavien–Dindo grade; Bold: statistically significant.

**Table 5:** Comparison of background data between open and minimally invasive AR for newly developed HCC.

	Before PSM			After PSM		
	OAR (N = 163)	MIAR (N = 113)	*p*	OAR (N = 76)	MIAR (N = 76)	*p*
Age, years	72 (31–91)	71 (21–86)	0.099	72 (43–86)	72 (29–85)	0.919
Sex, M/F	129/34	90/23	0.919	58/18	59/17	0.847
BMI, kg/m^2^	23.1 (14.7–54.0)	24.1 (16.0–36.3)	**0.011**	23.1 (14.7–54.0)	23.3 (16.0–36.3)	0.235
ASA score, I or II/≥III	125/38	103/10	**0.001**	64/12	67/9	0.481
Diabetes, n (%)	66 (40.5)	44 (38.9)	0.796	29 (38.2)	31 (40.8)	0.740
ICGR15, %	14.2 (0.6–52.6)	11.3 (0–39.4)	**0.0007**	12.6 (1.2–41.0)	12.1 (0–39.4)	0.227
≥13.0%, n (%)	89 (55.6)	40 (37.0)	**0.003**	35 (46.1)	36 (47.4)	0.871
Etiology, HBV/HCV/NBNC	32/56/75	23/37/53	0.961	16/27/33	14/25/37	0.803
Cirrhosis (histology), n (%)	49 (30.1)	41 (36.3)	0.278	31 (40.8)	31 (40.8)	1.000
Tumor characteristics						
Location, PS (%)/AL	119 (73.0)/44	71 (62.8)/42	0.073	53 (69.7)/23	55 (72.4)/21	0.721
Number	1 (1–23)	1 (1–4)	**0.049**	1 (1–23)	1 (1–4)	0.766
Single/Multiple	109/54	86/27	0.098	57/19	55/21	0.713
Size, cm	5.5 (1.2–22.0)	3.5 (0.7–16.0)	**<0.0001**	4.0 (1.2–22.0)	4.0 (0.7–16.0)	0.491
≥4.0 cm, n (%)	110 (67.5)	49 (43.4)	**<0.0001**	40 (52.6)	43 (56.6)	0.625
Stage, I/II/III/IVA/IVB	8/72/60/18/5	11/72/27/2/1	**0.0005**	6/42/24/3/1	8/42/23/2/1	0.973
AFP, ng/mL	15.9 (1.5–1,213,687.0)	7.3 (1.4–149,880.0)	**0.010**	13.7 (2.1–636,200.0)	9.8 (2.0–149,880.0)	0.340
DCP, mAU/mL	389 (3–538,983)	78 (10–47,453)	**0.002**	252 (3–538,983)	148 (10–47,453)	0.671
Differentiation			0.733			0.846
well	6	6		4	3	
moderate	150	102		70	69	
poor or sarcomatous	4	2		1	2	
combined	2	30		1	2	
necrosis	1	0		0	0	
Types of resection, n (%)			**0.0003**			0.905
Left lateral sectionectomy	5 (3.1)	7 (6.2)		4 (5.3)	3 (3.9)	
Segmentectomy	63 (38.7)	66 (58.4)		37 (48.7)	40 (52.6)	
Sectionectomy*	52 (31.9)	21 (18.6)		17 (22.4)	18 (23.7)	
≥Hemihepatectomy	43 (26.4)	19 (16.8)		18 (23.7)	15 (19.7)	
Major Hx (≥3 segs), n (%)	52 (31.9)	21 (18.6)	**0.014**	18 (23.7)	16 (21.1)	0.697

Continuous variables: median (range); PSM: propensity score matching; OAR and MIAR: open and minimally invasive anatomic liver resection, respectively; BMI: body mass index; ASA: American Society of Anesthesiology; ICGR15: the indocyanine green retention rate at 15 min; NBNC: non-B and non-C; PS: posterosuperior; AL: anterolateral; AFP: alpha-fetoprotein; DCP: des-gamma-carboxy prothrombin; Sectionectomy*: mono- or central bisectionectomy except for left lateral sectionectomy; Hx: hepatectomy; Bold: statistically significant.

**Table 6:** Comparison of background data between laparoscopic and robotic AR for newly developed HCC.

	Before PSM			After PSM		
	Laparoscopic AR (N = 89)	Robotic AR (N = 24)	*p*	Laparoscopic AR (N = 22)	Robotic AR (N = 22)	*p*
Age, years	70 (29–86)	72 (21–82)	0.975	71 (53–83)	72 (48–82)	0.778
Sex, M/F	71/18	19/5	0.948	19/3	19/3	1.000
BMI, kg/m^2^	24.0 (16.0–36.3)	24.2 (17.9–30.3)	0.744	23.5 (18.0–33.2)	24.4 (17.9–30.3)	0.411
ASA score, I or II/≥III	81/8	22/2	0.920	21/1	20/2	0.550
Diabetes, n (%)	37 (41.6)	7 (29.2)	0.269	9 (40.9)	7 (31.8)	0.531
ICGR15, %	11.3 (0.6–39.4)	11.3 (0–17.5)	0.329	10.5 (4.3–20.9)	11.2 (0.0–16.0)	0.865
≥13.0%, n (%)	32 (37.7)	8 (34.8)	0.801	6 (28.6)	7 (31.8)	0.817
Etiology, HBV/HCV/NBNC	18/30/41	5/7/12	0.912	5/6/11	4/7/11	0.910
Cirrhosis (histology), n (%)	38 (42.7)	3 (12.5)	**0.006**	3 (13.6)	3 (13.6)	1.000
Child-Pugh class, A/B	88/1	24/0	0.602	22/0	22/0	1.000
Tumor characteristics						
Location, PS (%)/AL	57 (64.0)/32	14 (58.3)/10	0.607	10 (45.5)/12	13 (59.1)/9	0.365
Number	1 (1–4)	1 (1–4)	0.517	1 (1–2)	1 (1–4)	0.936
Single/Multiple	69/20	17/7	0.495	17/5	17/5	1.000
Size, cm	3.5 (0.7–16.0)	3.1 (1.5–12.5)	0.111	3.3 (1.5–6.0)	3.1 (1.5–6.0)	0.814
≥4.0 cm, n (%)	43 (48.3)	6 (25.0)	**0.041**	5 (22.7)	5 (22.7)	1.000
Stage, I/II/III/IVA/IVB	11/57/19/1/1	0/15/8/1/0	0.252	5/13/4/0	0/14/7/1	0.077
AFP, ng/mL	8.8 (2.0–14,980.0)	6.0 (1.4–5,811.0)	0.185	5.4 (2.0–2,708.5)	6.0 (1.4–1,372.0)	0.890
DCP, mAU/mL	95 (10–47,453)	43 (14–20,843)	0.073	40 (10–2,753)	43 (14–20,843)	0.576
Differentiation			0.471			0.178
well	6	0		2	0	
moderate	79	23		20	22	
poor or sarcomatous	2	1		0	0	
combined	2	0		0	0	
Types of resection, n (%)			0.950			0.731
Left lateral sectionetomy	6 (6.7)	1(4.2)		1 (4.6)	0 (0)	
Segmentectomy	51 (57.3)	15 (62.5)		13 (59.1)	14 (63.6)	
Sectionectomy*	17(19.1)	4 (16.7)		5 (22.7)	4 (18.2)	
≥Hemihepatectomy	15 (16.9)	15 (16.9)		3 (13.6)	4 (18.2)	
Major Hx (≥3 segs), n (%)	17 (19.1)	4 (16.7)	0.786	3 (13.6)	4 (18.2)	0.680

Continuous variables: median (range); AR: anatomic liver resection; BMI: body mass index; ASA: American Society of Anesthesiology; ICGR15: the indocyanine green retention rate at 15 min; NBNC: non-B and non-C; PS: posterosuperior; AL: anterolateral; AFP: alpha-fetoprotein; DCP: des-gamma-carboxy prothrombin; Sectionectomy*: mono- or central bisectionectomy except for left lateral sectionectomy; Hx: hepatectomy; Bold: statistically significant.

**Table 7:** Details of recurrence after OAR or MIAR for newly developed HCC.

	Recurrent Cases in the Unmatched Cohorts			Recurrent Cases in the Matched Cohorts		
	OAR (N = 111)	MIAR (N = 51)	*p*	OAR (N = 50)	MIAR (N = 37)	*p*
Patterns of recurrence, n (%)			0.463			0.715
Intrahepatic-only	84 (75.7)	43 (84.3)		37 (74.0)	30 (81.1)	
Extrahepatic-only	7 (6.3)	2 (3.9)		3 (6.0)	2 (5.4)	
Intra- and extra-hepatic	20 (18.0)	6 (11.8)		10 (20.0)	5 (13.5)	
Extrahepatic recurrence, n (%)	27 (24.3)	8 (15.7)	0.215	13 (26.0)	7 (18.9)	0.438
Lung	13 (11.7)	5 (9.8)	0.720	6 (12.0)	5 (13.5)	0.834
Bone	11 (9.9)	2 (3.9)	0.193	6 (12.0)	1 (2.7)	0.115
Lymph node	9 (8.2)	2 (3.9)	0.325	5 (10.0)	2 (5.4)	0.436
Hematogenous metastasis	22 (19.8)	6 (11.8)	0.208	9 (18.0)	5 (13.5)	0.573
Recurrence-free survival (mo), range	7.6 (0.5–73.9)	15.4 (1.4–86.3)	**0.007**	9.4 (0.8–73.9)	15.4 (1.4–86.3)	0.125
First recurrence <1 year, n (%)	69 (62.2)	19 (37.3)	**0.003**	27 (54.0)	13 (35.1)	0.081
First recurrence <2 years, n (%)	87 (78.4)	34 (66.7)	0.111	38 (76.0)	24 (64.9)	0.257
Pathologic tumor stage, n (%)			**0.008**			0.696
I or II	49 (44.1)	34 (66.7)		29 (58.0)	23 (62.2)	
≥III	62 (55.9)	17 (33.3)		21 (42.0)	14 (37.8)	
Pathologic differentiation, n (%)			0.557			0.830
well	3 (2.7)	2 (3.9)		2 (4.0)	1 (2.7)	
moderate	104 (93.7)	46 (88.5)		46 (92.0)	33 (89.2)	
poor or sarcomatous	2 (1.8)	1 (1.9)		1 (2.0)	1 (2.7)	
combined	2 (1.8)	3 (5.8)		1 (2.0)	2 (5.4)	
Perioperative morbidity, n (%)						
Any (C–D grade ≥I)	57 (51.4)	21 (41.2)	0.229	25 (50.0)	16 (43.2)	0.533
Major (C–D grade ≥III)	18 (16.2)	6 (11.8)	0.459	5 (10.0)	5 (13.5)	0.612
Bile leak/collection	9 (8.1)	3 (5.9)	0.615	2 (4.0)	2 (5.4)	0.757
Treatment for recurrent tumor, n (%)						
Resection						
Any organs	35 (31.5)	23 (45.1)	0.094	16 (32.0)	15 (40.5)	0.411
Liver (n/with liver recurrence)	32/104 (30.8)	23/49 (46.9)	0.052	14/50 (29.8)	15/35 (42.9)	0.221
Use of MTA* or immunotherapy**	19 (17.7)	9 (17.1)	0.934	11 (22.0)	9 (24.3)	0.799

OAR: open anatomic liver resection; MIAR: minimally invasive anatomic liver resection; C–D: Clavien–Dindo classification; *MTA: molecular targeted agent; immunotherapy**: immunotherapy using immune checkpoint inhibitors; Bold: statistically significant.

## Data Availability

The datasets are not available for public access due to patient privacy concerns but are available from the corresponding author on reasonable request.
